# Diversity pattern of *Plasmodium knowlesi *merozoite surface protein 4 (MSP4) in natural population of Malaysia

**DOI:** 10.1371/journal.pone.0224743

**Published:** 2019-11-21

**Authors:** Md Atique Ahmed, Ahmed Saif, Fu-Shi Quan

**Affiliations:** 1 Department of Medical Zoology, School of Medicine, Kyung Hee University, Seoul, Republic of Korea; 2 Department of Clinical Laboratory Sciences, College of Applied Medical Sciences, Najran University, Najran, Saudi Arabia; 3 Medical Research Center for Bioreaction to Reactive Oxygen Species and Biomedical Science Institute, School of Medicine, Graduate school, Kyung Hee University, Seoul, Republic of Korea; Instituto Rene Rachou, BRAZIL

## Abstract

Human infections due to the monkey malaria parasite *Plasmodium knowlesi* are increasingly being reported from Malaysia. The parasite causes high parasitaemia, severe and fatal malaria in humans thus there is a need for urgent measures for its control. The MSP4 is a potential vaccine candidate, which is well studied in *Plasmodium falciparum* and *Plasmodium vivax*; however, no study has been conducted in the orthologous gene of *P*. *knowlesi*. In this study, we investigated the level of polymorphisms, haplotypes, natural selection and population structure of full-length *pkmsp4* in 32 clinical samples from Malaysian Borneo along with 4 lab-adapted strains. We found low levels of polymorphism across the gene with exon I showing higher diversity than the exon II. The C- terminal epidermal growth factor (EGF) domains and GPI-anchored region within exon II were mostly conserved with only 2 non-synonymous substitutions. Although 21 amino acid haplotypes were found, the frequency of mutation at the majority of the polymorphic positions was low. We found evidence of negative selection at the exon II of the gene indicating existence of functional constraints. Phylogenetic haplotype network analysis identified shared haplotypes and indicated geographical clustering of samples originating from Peninsular Malaysia and Malaysian Borneo. High population differentiation values were observed within parasite populations originating from Malaysian Borneo (Kapit, Sarikei and Betong) and laboratory-adapted strains obtained from Peninsular Malaysia and Philippines indicating distinct population structure. This is the first study to genetically characterize the full-length *msp4* gene from clinical isolates of *P*. *knowlesi* from Malaysia and thus would be very useful for future rational vaccine studies. Further studies with higher number of samples and functional characterization of the protein will be necessary.

## Introduction

*Plasmodium knowlesi*, a zoonotic malaria parasite of long-tailed and pig-tailed macaques is now considered as the fifth *Plasmodium* species infecting humans and now the most common cause of malaria in Malaysia. Most Southeast Asian countries have reported cases of this infection in humans [1,2]. As per the World Malaria Report 2017, there is a rapid increase of human cases in Malaysia [3] and within Malaysia, highest incidence of *knowlesi* malaria in human have been documented from Malaysian Borneo [4–6] highlighting the need of immediate and comprehensive approaches integrating multiple strategies such as vector control, anti-malarial treatment and development of effective vaccines. Almost 70–78% of malaria cases reported from Malaysian Borneo (Sarawak and Sabah) were due to *P*. *knowlesi* [6,7]. Death due to *P*. *knowlesi* malaria has also been reported in Sarawak and Sabah of Malaysian Borneo and rapid increase in parasitaemia has been shown to be associated with severe malaria and in some cases fatal in Malaysian Borneo [8,9]. Genetic studies and genomic studies on *P*. *knowlesi* clinical isolates from Malaysia have identified at least 3 sub-populations with their overall diversity even higher than *P*. *falciparum* and *P*. *vivax*, 2 of the populations were associated with primary primate hosts and one with geographical location [1,10–12]. Mitochondrial gene cytochrome oxidase I(cox 1) and the smaller subunit ribosomal rRNA (ssrRNA) of *P*. *Knowlesi* from clinical isolates and macaques also identified two distinct clusters which clustered geographically to Peninsular Malaysia and Malaysian Borneo [13].

One of the strategies to develop a vaccine against *Plasmodium* species is based on targeting apical organelle antigens or the merozoite surface antigens involved in the asexual-stage of the parasite life cycle, which are accessible to the host immune system [14]. Immune response induced by such antigens has the potential to block parasite entry into RBCs. However, because of high antigenic diversity (in field isolates), which is one of the main mechanisms through which the malaria parasites evade host immune responses remains as one of the challenges to design a strain-transcending vaccine. Major vaccine candidates studied till date in *P*. *falciparum* (like CSP, AMA1) show high polymorphism, evolve under positive natural selection and show high antibody response but rendered non-efficacious vaccine trial because of strain-specific immune response [15]. Merozoite surface protein family (MSPs) forms the most abundant protein, which are targets of immune attack by host antibodies and thus considered excellent targets for vaccine development. One of these proteins is the merozoite surface protein 4 (MSP4), an abundant glycosylphosphatidylinositol (GPI) anchored protein which contains a single epidermal growth factor (EGF)-like domain both these are towards the carboxyl terminus of the protein [16,17]. MSP4 has been considered as a promising subunit vaccine candidate in *P*. *falciparum* and naturally acquired antibody response hasbeen reported from malaria endemic regions [18,19].Vaccine trials in mouse models in *P*. *berghei* and *P*. *yoelli* have shown significant protective efficacy [20]. The structural conformation of the EGF-domain in *P*. *falciparum* MSP4 has been found to be essential for binding to host erythrocytes and antigenicity [21]. Recently, protective role of naturally acquired anti-PfMSP4 antibodies was found to be associated with clinical malaria in an endemic region of Senegal [22] supporting further development of MSP4 as a vaccine candidate. Genetic studies in both *P*. *falciparum* and *P*. *vivax* MSP4 gene have been extensively conducted in different endemic areas of the world and the gene is found to possess low level of polymorphism and under purifying selection[23–25]. Vaccine studies in *P*. *knowlesi* are still in its nascent stage. High genetic diversity has been observed in clinical isolates of *P*. *knowlesi* in several ortholog vaccine antigens (like NBPXA, MSP1 and MSP7D)[11,26–28]. However, no genetic study has been done in the *pkmsp4* gene from clinical isolates of Malaysia.

In this study, 36 *pkmsp4* full-length sequences (32 clinical isolates from Sarawak, Malaysian Borneo and 4 long-time lab-adapted strains) were obtained from published genome studies and the level of nucleotide diversity, haplotypes, and natural selection acting at full-length MSP4 gene were determined. Information on genetic diversity and natural selection acting at *msp4* gene will be essential for a rational approach for vaccine design and functional assays.

## Materials and methods

### *pkmsp4* sequence data

*pkmsp4* sequence data were obtained from genomes of 32 clinical samples originating from Sarawak, Malaysian Borneo obtained from a previous genome study along with 4 long-time isolated lines originated from Peninsular Malaysia and Philippines (along with the H-strain, PKNH_0414100)[1,10] which were orthologous to *P*. *vivax* (PVX_003775). These 4 long-time isolated laboratory lines were maintained in rhesus macaques which were originally obtained from Peninsular Malaysia and Philippines in 1960 [10]. The original genome study was conducted with appropriate informed consent from patients and with clearance from ethical committees [10]. The accession numbersof the sequences along with the location of sample collection are listed in S1 Table. The genomes were downloaded from the European Nucleotide Archive (https://www.ebi.ac.uk/ena). Sequence data were aligned using the CLUSTAL-W program in MegAlignLasergene v 7.0 (DNASTAR). The signal peptide within the full-length PkMSP4 amino acid sequence was predicted using Signal IP-5.0 software [29].

### Sequence diversity and natural selection

Sequence diversity (π), which is defined as the average number of nucleotide differences per site between two sequences was determined by DnaSP v5.10 software. Number of polymorphic sites, singleton sites (a nucleotide variant that appears only once in among the sequences), number of synonymous (silent mutations) and non-synonymous substitutions (replacement mutations or mutations leading to change in amino acids), number of haplotypes (H) and haplotype diversity (Hd) within the *pkmsp4* sequences were determined by DnaSP v5.10 software [30].

Natural selection was determined at the intra and inter-species levels. At the intra-population level, natural selection was determined by calculating the rates of synonymous substitutions per synonymous site (dS) and non-synonymous substitutions per non-synonymous site (dN) as computed by using Nei and Gojobori’s method and robustness was estimated by the bootstrap method with 1000 pseudo replicates as implemented in the MEGA 5.0 software[31]. Difference between dN and dS was determined by applying codon based Z-test (*P <*0.05) in MEGA software v.5 with 1000 bootstrap replications [31]. The Tajima’s D, Fu & Li’s D* and F* neutrality tests were performed as implemented in DnaSP v5.10 software. Tajima’s D is expected to be 0 for a gene which is not under the influence of any selection pressure. When Tajima’s D values are positive and significant, it indicates positive/balancing selection, whereas negative values suggest negative selection or population expansion. Significant positive values for Fu & Li’s D* and F* also indicate population contraction due to a selection event while negative values indicate population expansion and excess of singletons. To test whether the *pkmsp4* gene is under the influence of natural selection in the inter-species level, the robust McDonald and Kreitman(MK) test was performed with *P*. *coatneyi* (PCOAH_00008580) *msp4* gene as an out-groups using DnaSP v5.10 software[30]. Graphical representation of nucleotide diversity and Tajima’s D across the full-length *pkmsp4* genes were conducted using the same software with window length 100 and step size 50 bp using.

### Haplotype network analysis

Genealogical relationships between the *pkmsp4* nucleotide haplotypes were constructed using the median-joining method with default parameters in NETWORK software (version 4.6.1.2, FluxusTechnology Ltd, Suffolk, UK). The analysis aimed to reconstruct haplotype networks of the entire set of *P*. *knowlesi msp4* genes, with color-coded haplotypes for geographical origins. Straight lines connect pairs of haplotypes that differ by a single mutational step.

### Genetic differentiation

The ARLEQUIN software v.3.5.1.3 [32] was used to compute pairwise differences (*F*_*ST*_) between *P*. *knowlesi* populations from four different geographical locations of Malaysia i.e. Kapit, Sarikei, Betong and the laboratory-adapted strains. The *F*_*ST*_ values were determined with 10,100 permutations. *F*_*ST*_ is a comparison of the sum of genetic variability within and between populations based on the differences in allelic frequencies. *F*_*ST*_ values are interpreted as no (0), low (>0–0.05), moderate (0.05–0.15), and high (0.15–0.25) genetic differentiation.

## Results

### Schematic structure and polymorphism within PkMSP4

**Fig 1:**
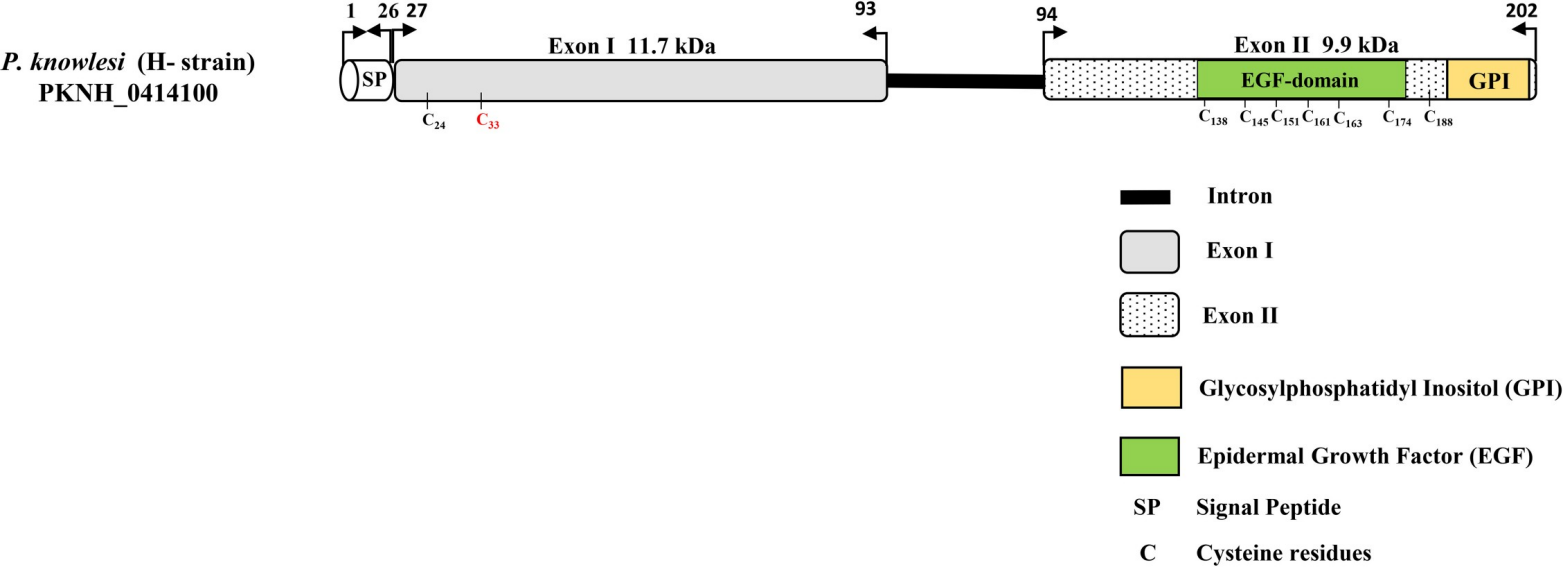
Schematic diagram of *Plasmodium knowlesi* MSP4 protein (PKNH_0414100, 202 amino acid sequence). Each box in the schematic diagram is representative of exon I and exon II and the EGF and the GPI-anchored regions within are marked. The six Cystines within the EGF domains are marked with amino acid positions in the subscript. A cysteine residue that was found only among the clinical isolates of Sarawak, Malaysian Borneo are marked in red. Exon1 and exon II along with its molecular weight, are in shaded and dotted background, respectively. Signal peptide is abbreviated as (SP).

**Fig 2:**
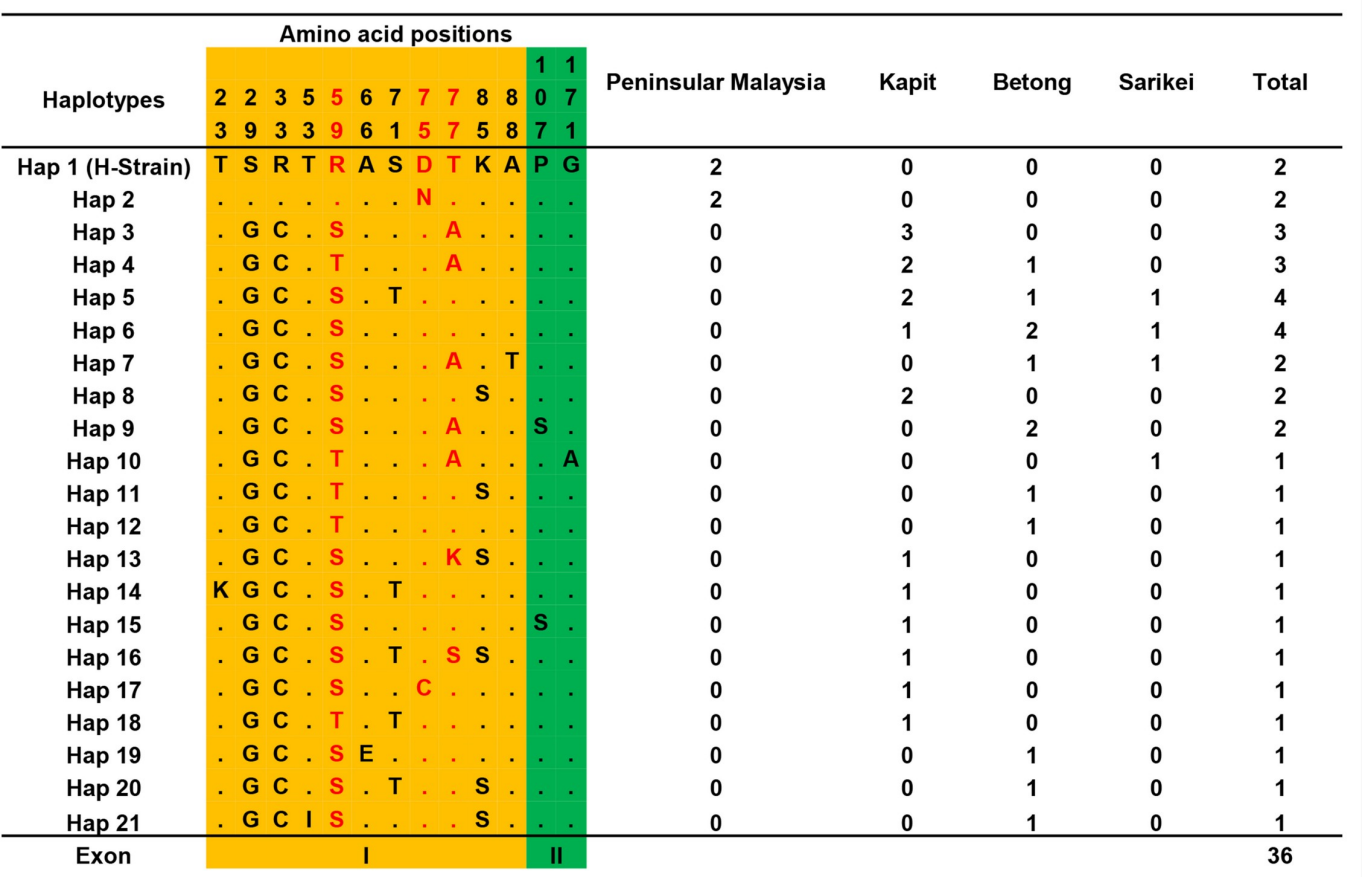
Amino acid haplotypes found within 36 PkMSP4 sequences. Amino acids identical to reference strain H (PKNH_0414100) are marked in the top row (Hap1). The shaded regions i.e. yellow and green represents exon I and exon II respectively. Amino acids marked in red represent the hypervariable amino acids. Total number of sequences for each haplotype and their origin are listed in the right panel. Dots represent identical amino acids and the number in vertical represents the position of the amino acids with respect to the H-strain.

The schematic structure of the PkMSP4 protein based on the H-strain with 2 exons (Exon I, 280 bp, and Exon II, 235 bp), C-terminal single EGF-domain and GPI-anchored region is described in Fig 1. The signal peptide of the PkMSP4 protein was detected between amino acid positions 22 to 26 by the SignalP server S1 Fig. Alignment and comparison of deduced amino acid sequences between the lab-adapted strains (including the H-strain) and the clinical isolates from Sarawak showed that there was an amino acid substitution from arginine to cysteine(R33C) in all the clinical isolates (n = 34) from Sarawak Fig 1. All six cysteine residues within the EGF-domain of exon II were conserved in the clinical isolates as well in the lab-adapted strains from Peninsular Malaysia and Philippines. Within the exon I, there were three hyper-variable amino acid polymorphisms R59S/T, D75N/C and T77A/S Fig 2. Amino acid alignment of 36 isolates identified 21 haplotypes Fig 2. The region-wise distribution map of the haplotypes indicated that haplotypes from Peninsular Malaysia (Hap 1 and Hap 2) were distinct from rest of clinical isolates from Malaysian Borneo Fig 2. The EGF-domains were mostly conserved with only one single amino acid change G171A in one isolate from Sarikei (Hap 10) Fig 2. Shared amino acid haplotypes (Hap 5 and Hap 6) were noted only between samples from Malaysian Borneo. The GPI-anchored region was completely conserved in all 36 isolates. The amino acid polymorphisms within 36 isolates across the full-length PkMSP4 protein are shown in S2 Fig.

### Nucleotide diversity and polymorphisms

**Fig 3:**
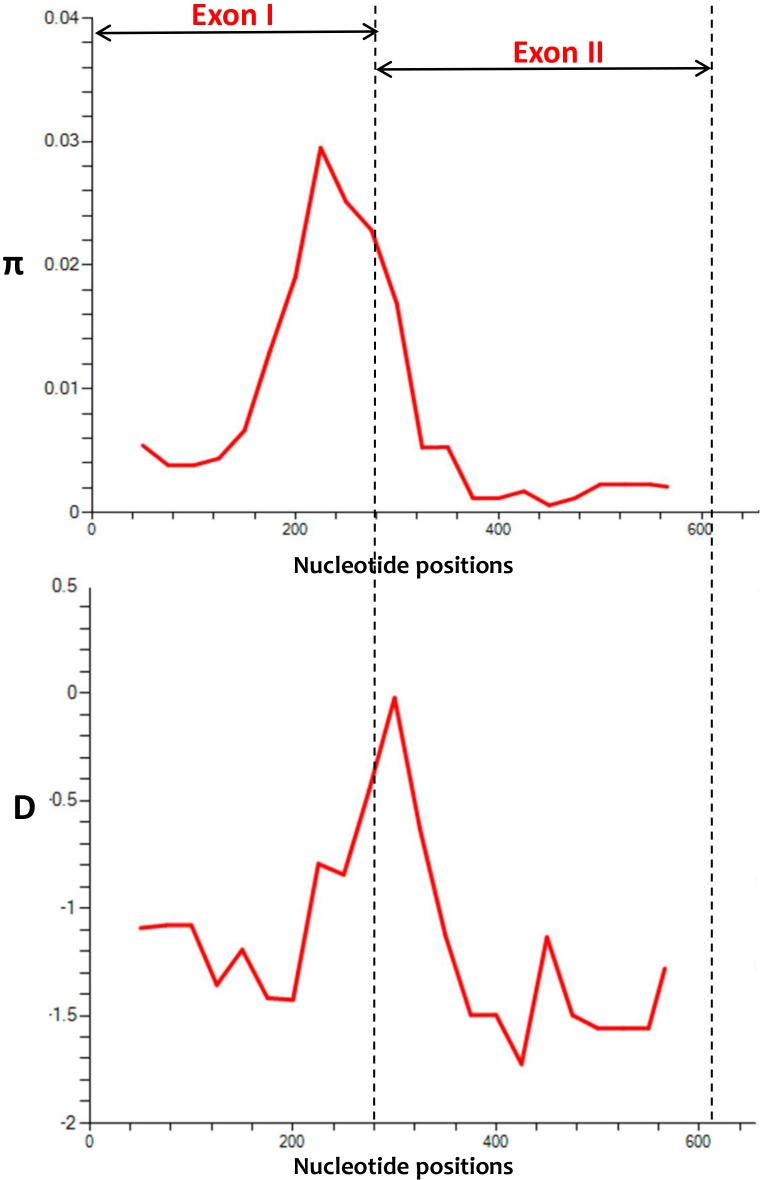
(A) Graphical representation of nucleotide diversity (π) within 36 full-length *pkmsp4* genes (606 bp) from Malaysia. The exons are marked and with double-sided arrows. (B) Graphical representation of Tajima’s D value across the *pkmsp4* gene. Dotted lines are used to indicate the peaks in the π graph and D graph.

Analysis of nucleotide alignment of 36 full-length *pkmsp4* sequences (606 bp) revealed that there were 29 (4.7%) polymorphic sites, of which 13 were singleton sites and 16 were parsimony informative sites. The overall nucleotide diversity across the full-length gene was π = 0.007 ± SD 0.000 Table 1 which was higher compared to MSP4 orthologs in *P*. *vivax* and *P*. *falciparum* [23,25]. There were 29 SNPs (17 non-synonymous and 12 synonymous substitutions) across the full-length gene (Table 2). These 29 SNPs led to 24 nucleotide haplotypes and the haplotype diversity was 0.975, which was higher compared to exon I and exon II (Table 1). The sliding window analysis of nucleotide diversity across the full-length gene also indicated higher level of diversity in exon I compared to exon II which constituted the single EGF domain and the GPI-anchored region (Fig 3A). The diversity ranged from 0 to 0.28 while the higher values were towards the exon I Fig 3A.

**Table 1:** Estimates of nucleotide diversity, natural selection, haplotype diversity and neutrality indices of *pkmsp 4*.

Domain	No. samples	SNPs	No. haplotype	Diversity ± SD		d_N_-d_S_	Codon based *z* test	Taj D	Fu & Li’s D*	Fu & Li’s F*
				Haplotype	Nucleotide					
Full-length	36	29	24	0.975± 0.013	0.007± 0.000	-0.96	*P* > 0.1	-1.40	-1.65	-1.85
Exon I		21	19	0.946± 0.009	0.012± 0.001	0.23	*P* > 0.1	-1.11	-1.37	-1.51
Exon II		9	10	0.664± 0.010	0.002± 0.005	-2.04	*P* < 0.01	-1.73	-1.66	-1.97

SNPs; single nucleotide polymorphisms, SD; Standard deviation

**Table 2:** Synonymous and non-synonymous sites of *pkmsp4*.

Location	n	SNPs	Syn.	Non-syn.
Full-length	36	29	12	17^a^
Exon I		21	5	16 ^b^
Exon II		9	7	2

SNPs Single nucleotide polymorphisms; Syn. Synonymous substitutions; Non-syn. Nonsynonymous substitutions; n number of samples

^a, b^; includes sites with complex codons

Domain wise analysis of the exons indicated that exon I had higher number (SNPs = 21) of polymorphic sites of which 16 non-synonymous (including five complex sites) and 5 synonymous sites, compared to exon II; SNPs = 9 (2 non-synonymous and 7 synonymous) sites (Table 2 and S3 Fig). Within exon I there were five SNPs which were variable at the second and third base of a codon (complex codons) (G176C, A177C and A229G, C230A) which led to hyper-variable amino acids at positions R59S/T, D75N/C and T77A/K/S (Fig 2). The higher number of SNPs in exon I (with 8 singleton sites) led to a higher number of haplotypes, haplotype diversity and nucleotide diversity (H = 19, Hd = 0.946 and π = 0.012) compared to exon II (Table 1).

### Natural selection

To determine whether natural selection contributes to the polymorphism in the *pkmsp4* full-length gene and at each exon, multiple tests were conducted both at the inter-as well as intra-species levels. At the intra-species level, the full-length genes showed negative values were obtained for dN-dS, Tajima’s D and Fu and Li’s D* and F* values (Table 1) indicating purifying/negative selection and population expansion. However, values obtained were not significant. Independent tests for both exon I and II also showed similar results except for exon I which showed positive values for dN-dS = 0.23 (Table 1). Indeed this was obvious because of higher number of non-synonymous substitutions in Exon I (Table 2), but most of these were due to low frequency singleton variable sites indicating negative natural selection and parasite population expansion. At the inter-species level, the robust MK test was performed with *P*. *coatneyi msp4* gene as outgroup sequence. Test results showed significant negative natural selection for exon II (NI = 0.105, *P < 0*.05) which contained the EGF-domain and the GPI-anchored regions, whereas as for the full-length *pkmsp4* gene it showed neutrality index of 0.418 *P = 0*.*06* indicating overall negative/purifying selection (Table 3). However, MK test for exon I showed NI = 1.28 but not significant (Table 3). Sliding window plot analysis of Tajima’s D across the full-length *pkmsp4* gene also indicated most values below 0 indicating purifying selection, however SNPs from 280–320 showed positive D values. (Fig 3B).

**Table 3:** McDonald–Kreitman tests on MSP4 of *Plasmodium knowlesi* with *P*. *coatneyi* as outgroup species.

MSP4	Polymorphic changes within species		Fixed differences between species		Neutrality Index
	Syn.	NonSyn.	Syn.	NonSyn.	
Full-length	12	17	24	73	0.418^¥^
Exon I	5	16	18	45	1.280
Exon II	7	2	14	38	0.105**

**Fisher’s exact test P-value *< 0*.*005*

^¥^ Fisher’s exact test P-value = *0*.*06*

Syn. Synonymous sites; NonSyn.; Non synonymous sites

### Haplotype network analysis

**Fig 4:**
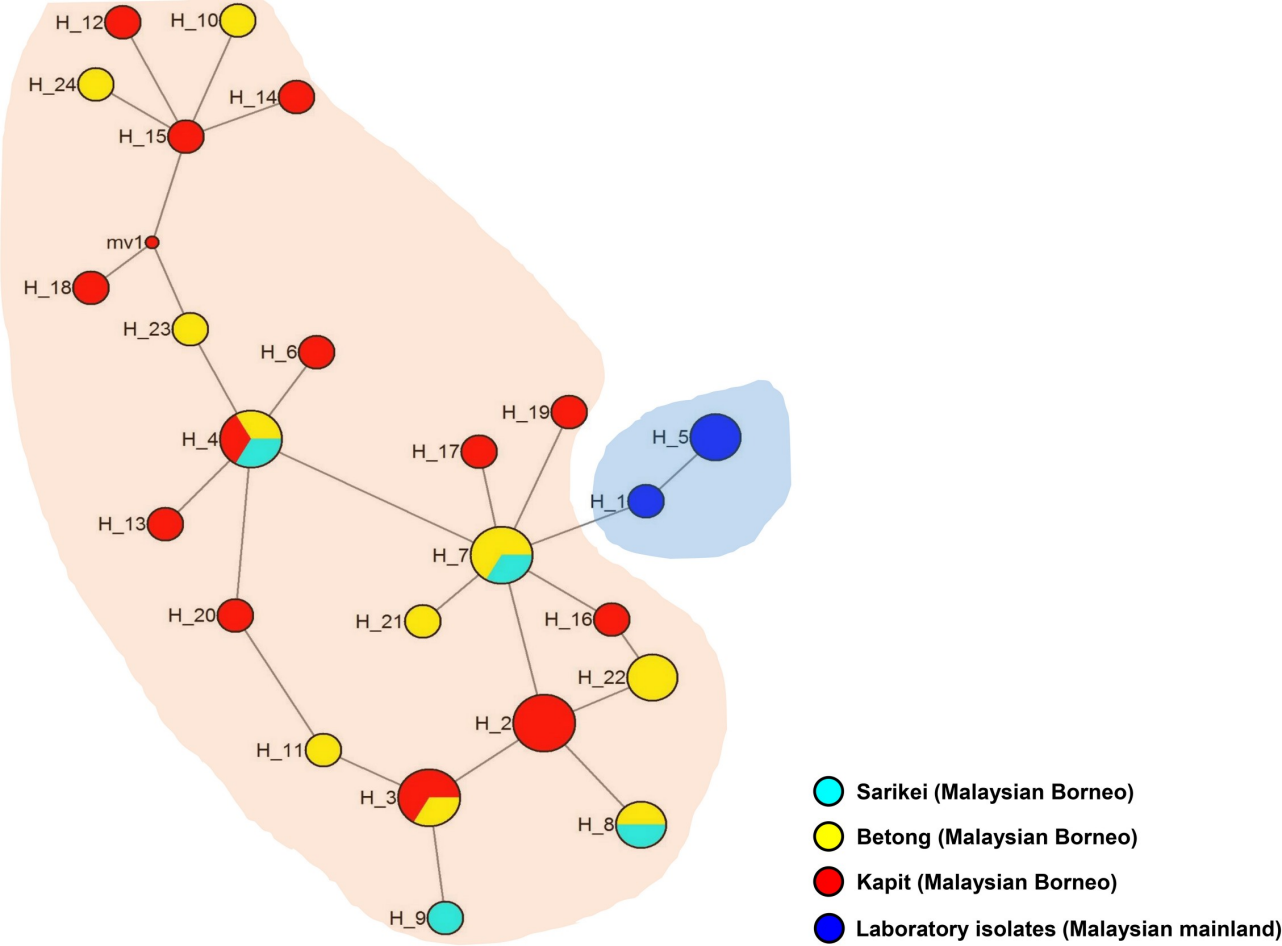
Median-joining networks of *Plasmodium knowlesi* MSP4 nucleotide haplotypes from Malaysia. The genealogical haplotype network shows the relationships among the 24 haplotypes present in the 36 nucleotide sequences obtained from clinical isolates of Sarawak, Malaysian Borneo and laboratory adapted strains. Each distinct haplotype has been designated a number (H_n). Circle sizes represent the frequencies of the corresponding haplotype (the number is indicated for those that were observed >1×). Distances between nodes are arbitrary. The small red circles are median vectors (marked mv1) randomly generated by the software while constructing the network.

Genealogical haplotype network analysis identified two distinct population clusters based on the geographical origin; one cluster originating in the laboratory-adapted strains (i.e. H, Malayan, Philippine and MR4H) and the other sub-cluster was the clinical isolates from Sarawak, Malaysian Borneo (Fig 4). Shared haplotypes between parasite populations from Kapit, Betong and Sarikei (H_4) (Fig 4) indicating a common origin of parasites from the region. Similar findings with clinical isolates from Malaysian Borneo have been reported earlier with other merozoite surface proteins [26,28].The 24 *pkmsp4* nucleotide haplotypes identified in this study are listed in S2 Table.

### Genetic differentiation within *P*. *knowlesi* populations

Pairwise population differentiation index (*F*_*ST*_ values) using ARLEQUIN software identified very high and significant genetic differentiation within parasite populations originating from Malaysian Borneo (Kapit, Sarikei and Betong), Malaysian Borneo and laboratory-adapted strains (*F*_*ST*_ = 0.53–0.66, *P < 0*.*05*) Table 4. These *F*_*ST*_ values suggests that parasitic transmission is confined to these geographically distinct regions i.e. Peninsular Malaysia and Malaysian Borneo. Genetic differentiation of parasite populations originating from Sarawak, Malaysian Borneo (i.e. Kapit, Sarikei and Betong) were low (*F*_*ST*_ = 0.03–0.07, *P > 0*.*05*) Table 4 indicating localized transmission within Sarawak.

**Table 4:** Population differentiation values (*F*_*ST*_) from each parasite populations of Malaysia based on *Pkmsp4* genes.

Location	*F*_*ST*_ values			
	Peninsular Malaysia	Kapit (Sarawak)	Betong (Sarawak)	Sarikei (Sarawak)
Peninsular Malaysia	-			
Kapit (Sarawak)	0.537**	-		
Betong (Sarawak)	0.571**	0.040	-	
Sarikei (Sarawak)	0.666**	0.037	0.070	-

** P < 0.05

## Discussion

*P*. *knowlesi* has gained substantial research interest in recent years as a high proportion of human cases specifically from Malaysia and most Southeast Asian countries have been reported and it can cause high parasitemia in humans which in certain cases become severe disease and can be fatal [1]. Blood stage antigens localized at the merozoite surface play an important role in invasion into erythrocytes and these antigens are directly exposed to host immune response during merozoite egress and thus are considered excellent vaccine candidates. A candidate antigen should optimally possess low polymorphism to be efficacious across different geographical locations and avoid allele-specific immune response. Merozoite surface proteins (MSPs), specifically MSP4 is recognized as a potential vaccine candidate for *P*. *falciparum* as it has been found to elicit a strong antibody response in patients and bound to RBCs [21,22]. Thus, in this study, we studied the level of polymorphism and natural selection of *msp4* from clinical isolates of *P*. *knowlesi* from Sarawak, Malaysian Borneo and lab-adapted strains from Peninsular Malaysia and Philippines.

We found low levels of polymorphism (SNP = 29) across the full-length *pkmsp4* gene and the majority of the SNPs were localized toward the exon I. There were only 2 non-synonymous substitutions in exon II (P107S and G171A), of which the latter was a single amino acid change present in only one isolate from Malaysian Borneo indicating conserved function within the single EGF-domain. The 6 cysteine residues within the EGF-domain were conserved within the 36 isolates (including the lab adapted strains) indicating conserved functional activity. It is interesting to note that *pvmsp4* and *pfmsp4* show a similar pattern of higher polymorphism in exon I compared to exon II [23,24,33]. The GPI-anchored region was completely conserved in *pkmsp4* clinical isolates from Sarawak and similar conservation was observed in *pfmsp4* and *pvmsp4* and these anchored regions were found to be essential for proper folding and immunogenicity of the whole protein [21]. The PkMSP4 protein did not possess any tandem repeat units as observed for PvMSP4 and PfMSP4 proteins [23,24]. Among all the MSPs studied till date in *P*. *knowlesi*, low polymorphisms towards the C-terminal EGF-domains have been shown in only a few antigens example *msp1p* [34].

We observed negative/purifying selection within the 36 *pkmsp4* genes from Malaysia. We verified natural selection tests both at the inter- and intra-species levels and found significant tests results for the exon II which constituted the EGF and GPI-anchored domains indicating functional constraints. The natural selection results obtained in this study indicate that *msp4* gene may not be exposed to host immune pressure however, graphical representation of Tajima’s D values indicated some SNPs with positive values within Exon II. Thus, immunological and functional characterization would be necessary as ortholog in *P*. *vivax* and *P*. *falciparum* elicit a strong immune response in patient sera[19,22]. Haplotype data from both amino acid and nucleotide sequences showed a similar pattern of clustering of isolates based on geographical origin i.e. from Peninsular Malaysia and Malaysian Borneo. Indeed, the haplotype network tree generated using the 24 nucleotide haplotypes showed geographical clustering more clearly, as observed for other MSPs of *P*. *knowlesi*[34].

Population differentiation index *F*_*ST*_based on *pkmsp4* showed high genetic differentiation values between the long-term isolated laboratory-adapted strains and Sarawak, Malaysian Borneo and this was due to the geographical distance between the two regions, which is separated by the South China Sea. These results indicate localized transmission in Sarawak, Malaysian Borneo. However, a vaccine designed based on the low polymorphic *pkmsp4* domains could still be effective for all sub-populations. Thus, further characterization through genetic as well as immunological studies is necessary.

## Conclusions

The present study is the first to investigate genetic diversity and natural selection of the *pkmsp4* gene from clinical samples of Sarawak and laboratory-adapted strains. Low level of genetic diversity was observed across the gene with only two non-synonymous substitutions within the EGF-domain. Overall, the gene was under negative/purifying natural selection, however, certain regions in Exon II showed high Tajima’s D values thus could be under balancing selection. Distinct population structure was observed and high genetic differences between parasites populations originating from Peninsular Malaysia and Malaysian Borneo was noted indicating absence of gene flow between the two regions. Further genetic studies with a higher number of clinical isolates (specifically form Peninsular Malaysia) as well as immunological studies characterizing the functional domains would be necessary to validate *pkmsp4* as a potential vaccine candidate for *P*. *knowlesi*.

## Supporting information

S1 Table Accession number of *pkmsp4* sequences used in the study and their geographical origin. (DOCX)

S2 Table List of nucleotide haplotypes identified with *pkmsp4*. (DOCX)

S1 Fig Signal peptide predicted with cleavage site positions between 22 to 26 amino acids. (TIF)

S2 Fig Amino acid polymorphism within 36 PkMSP4 sequences. Yellow and green shaded regions represent exon I and II respectively. The amino acid positions are marked on top as numbers based on the H-strain and the red colored amino acid represents hypervariable amino acids.
(TIF)

S3 Fig Nucleotide polymorphism within 36 *pkmsp4* sequences. Yellow and green shaded regions represent exon I and II respectively.
(TIF)
