# Supplementary material for: Diversity pattern of Plasmodium knowlesi merozoite surface protein 4 (MSP4) in natural population of Malaysia
Source: PLoS One. 2019 Nov 21;14(11):e0224743. doi: 10.1371/journal.pone.0224743 (PMC6872184; doi:10.1371/journal.pone.0224743)
Supplement: S1 Table — (DOCX) [file pone.0224743.s001.docx]

Supplementary Table S1. Study samples and origin

| No. | Sample | Area | Year |
| --- | --- | --- | --- |
| 1 | ERR274221 | Sarikei(MB) | 2012/3 |
| 2 | ERR274222 | Sarikei(MB) | 2012/3 |
| 3 | ERR366425 | Sarikei(MB) | 2012/3 |
| 4 | ERR366426 | Sarikei(MB) | 2012/3 |
| 5 | ERR985374 | Betong(MB) | 2012/3 |
| 6 | ERR985376 | Betong(MB) | 2012/3 |
| 7 | ERR985377 | Betong(MB) | 2012/3 |
| 8 | ERR985378 | Betong(MB) | 2012/3 |
| 9 | ERR985379 | Betong(MB) | 2012/3 |
| 10 | ERR985380 | Betong(MB) | 2012/3 |
| 11 | ERR985381 | Betong(MB) | 2012/3 |
| 12 | ERR985382 | Betong(MB) | 2012/3 |
| 13 | ERR985383 | Betong(MB) | 2012/3 |
| 14 | ERR985384 | Betong(MB) | 2012/3 |
| 15 | ERR985385 | Kapit(MB) | 2012/3 |
| 16 | ERR985387 | Kapit(MB) | 2012/3 |
| 17 | ERR985388 | Kapit(MB) | 2012/3 |
| 18 | ERR985390 | Kapit(MB) | 2012/3 |
| 19 | ERR985392 | Kapit(MB) | 2012/3 |
| 20 | ERR985393 | Kapit(MB) | 2012/3 |
| 21 | ERR985394 | Kapit(MB) | 2012/3 |
| 22 | ERR985397 | Kapit(MB) | 2012/3 |
| 23 | ERR985404 | Kapit(MB) | 2012/3 |
| 24 | ERR985406 | Kapit(MB) | 2012/3 |
| 25 | ERR985407 | Kapit(MB) | 2012/3 |
| 26 | ERR985408 | Kapit(MB) | 2012/3 |
| 27 | ERR985409 | Kapit(MB) | 2012/3 |
| 28 | ERR985410 | Betong(MB) | 2012/3 |
| 29 | ERR985411 | Betong(MB) | 2012/3 |
| 30 | ERR985417 | Kapit(MB) | 2012/3 |
| 31 | ERR985418 | Kapit(MB) | 2012/3 |
| 32 | SRR2225571 (MR4) | P. Malaysia |  |
| 33 | SRR2225573 (Philippine) | P. Malaysia |  |
| 34 | Malayan Strain Pk1A [PKNOH_S08495000](https://plasmodb.org/plasmo/app/record/gene/PKNOH_S08495000) | P. Malaysia |  |
| 35 | H-strain(PKNH_0414100) | P. Malaysia |  |
| 36 | ERR985389 | Kapit(MB) | 2012/3 |

P; Peninsular, MB; Malaysian Borneo
