# Supplementary material for: Diversity pattern of Plasmodium knowlesi merozoite surface protein 4 (MSP4) in natural population of Malaysia
Source: PLoS One. 2019 Nov 21;14(11):e0224743. doi: 10.1371/journal.pone.0224743 (PMC6872184; doi:10.1371/journal.pone.0224743)
Supplement: S2 Table — (DOCX) [file pone.0224743.s002.docx]

| Haplotype number | Haplotype |
| --- | --- |
| H_1 | GCACTCGACGGGAACTAATGGCCCCTGGT |
| H_2 | ACGTTCGCCGGGAGCTAATGGCCCCTGGT |
| H_3 | ACGTTCCCCGGGAGCTAATGGTCCCTGGT |
| H_4 | ACGTTCGCCGCGAACTAATGGCCCCTGGT |
| H_5 | GCACTCGACGGAAACTAATGGCCCCTGAT |
| H_6 | ACGTTCGCCGCGAACGAATGGCCCCTGGT |
| H_7 | ACGTTCGCCGGGAACTAATGGCCCCTGGT |
| H_8 | ACGTTCGCCGGGAGCTAATAGCCCCTGGT |
| H_9 | ACGTTCCCCGGGAGCTAATGGTCCCTCGT |
| H_10 | ACGTTCCCCGGGAACTTCCGGCCCCTGGT |
| H_11 | ACGTTCCCCGGGAACTAACGGTCCCTGGT |
| H_12 | ACGTTCGCCGGGAAATTCCGGCCCTTGGT |
| H_13 | AAGTTCGCCGCGAACTAATGACCCCTGGT |
| H_14 | ACGTTCGCCGGGAACTTCCGGCCCCTGGC |
| H_15 | ACGTTCGCCGGGAACTTCCGGCCCCTGGT |
| H_16 | ACGTTCGCCGGGAACTAATGGCTCCTGGT |
| H_17 | ACGTTCGCCGGGAACTAATGGCCCCCGGT |
| H_18 | ACGTTCGCCGCGATCTTCCGACCTCTGGT |
| H_19 | ACGTTCGCCGGTGACTAACGGCCCCTGGT |
| H_20 | ACGTTCCCCGCGAACTAACGGCCCCTGGT |
| H_21 | ACGTTCGCAGGGAACTAATGGCCCCTGGT |
| H_22 | ACGTTCGCCGGGAGCTAATGGCTCCTGGT |
| H_23 | ACGTTCGCCGCGAACTTCTGGCCCCTGGT |
| H_24 | ACGTCTGCCAGGAACTTCCGGCCCCTGGT |

S2 Table: List of nucleotide haplotypes identified with 36 *pkmsp4* sequences
