# Supplementary figures and images for: Diversity pattern of Plasmodium knowlesi merozoite surface protein 4 (MSP4) in natural population of Malaysia
Source: PLoS One. 2019 Nov 21;14(11):e0224743. doi: 10.1371/journal.pone.0224743 (PMC6872184; doi:10.1371/journal.pone.0224743)

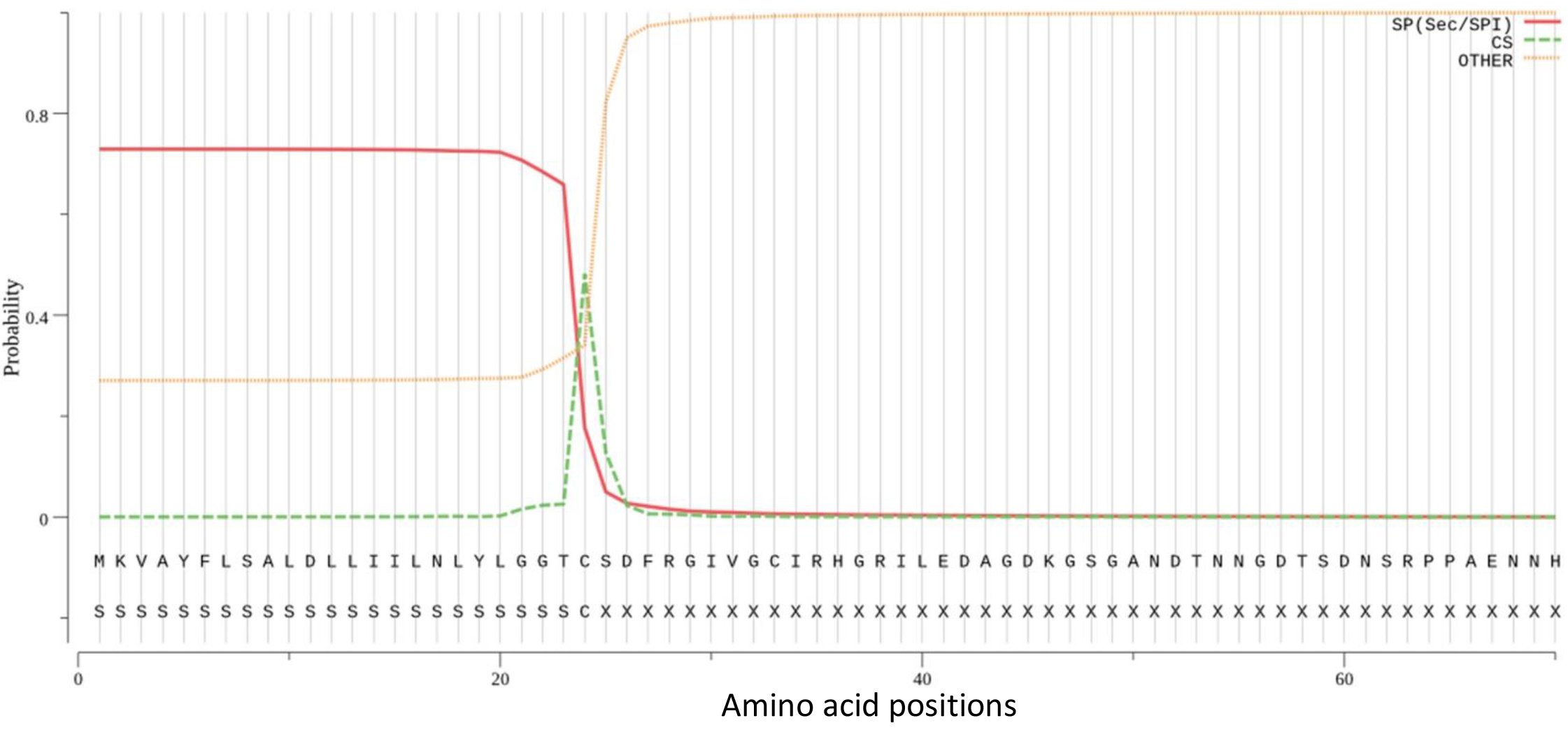

Supplement: S1 Fig — (TIF) [file pone.0224743.s003.tif]

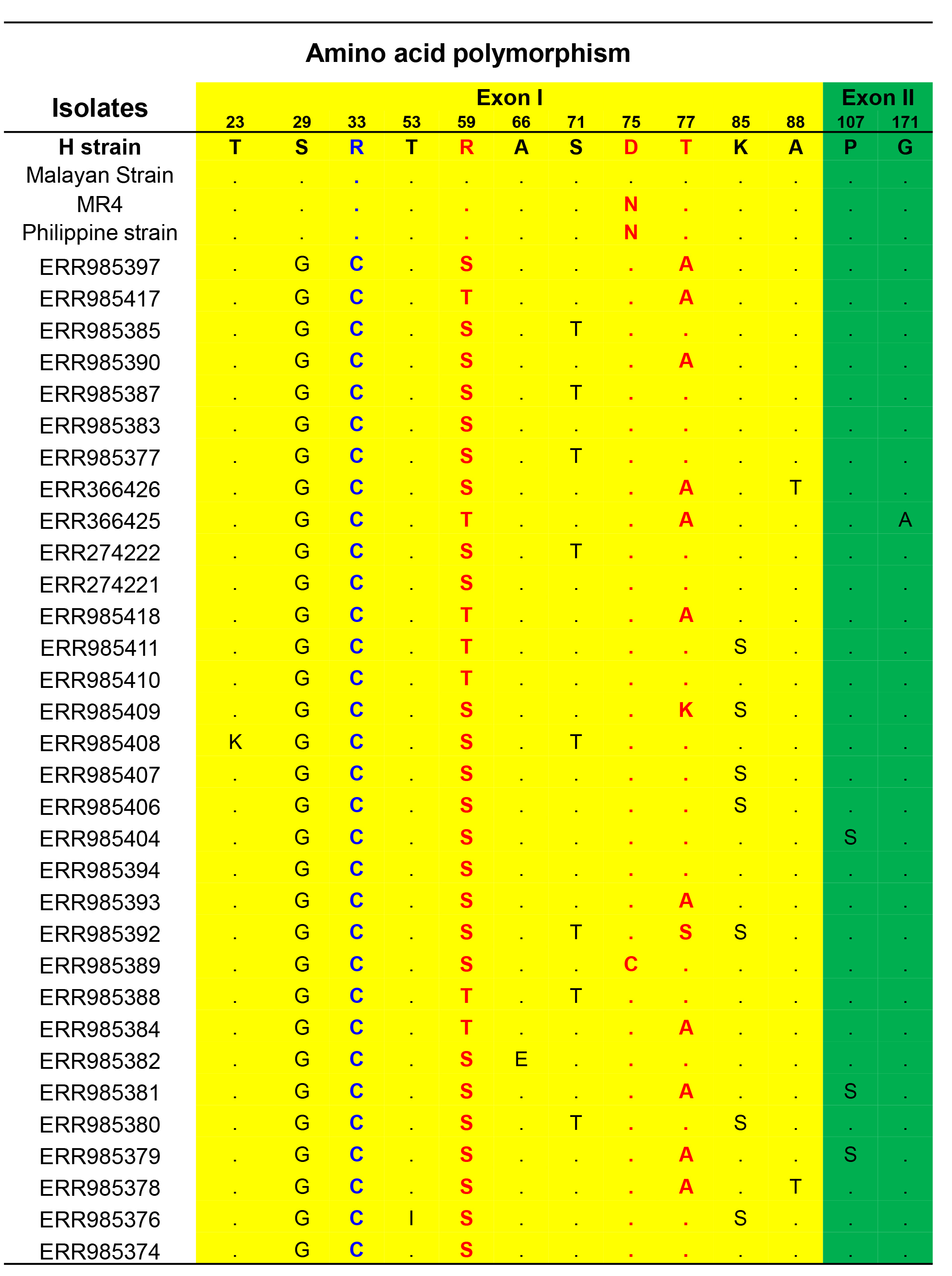

Supplement: S2 Fig — Yellow and green shaded regions represent exon I and II respectively. The amino acid positions are marked on top as numbers based on the H-strain and the red colored amino acid represents hypervariable amino acids. (TIF) [file pone.0224743.s004.tif]

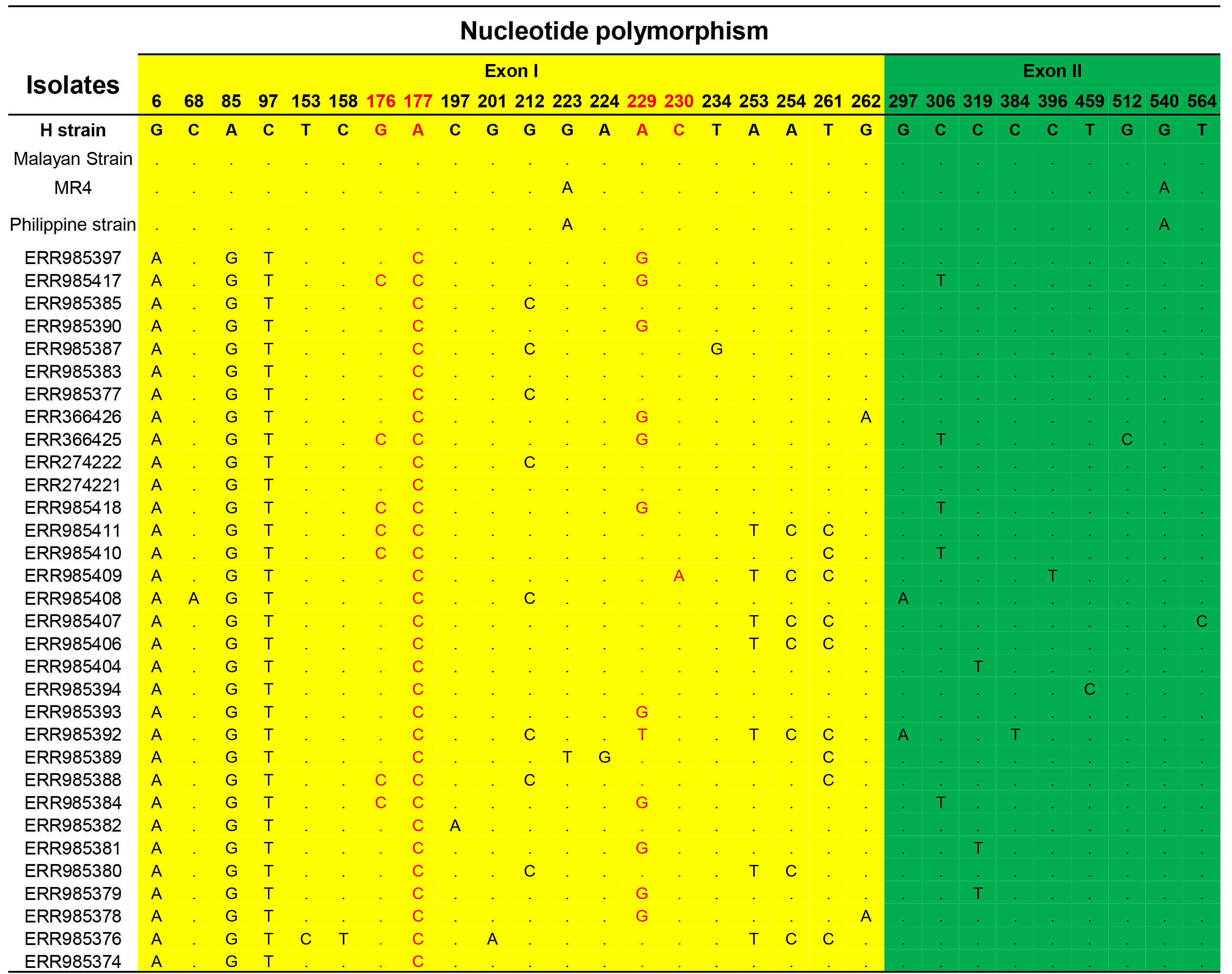

Supplement: S3 Fig — Yellow and green shaded regions represent exon I and II respectively. (TIF) [file pone.0224743.s005.tif]
